# Supplementary material for: The added value of family-centered rounds in the hospital setting: A systematic review of systematic reviews
Source: PLoS One. 2023 Jan 20;18(1):e0280142. doi: 10.1371/journal.pone.0280142 (PMC9858825; doi:10.1371/journal.pone.0280142)
Supplement: S1 Appendix — (DOCX) [file pone.0280142.s002.docx]

Search strategy

PUBMED

(“Teaching Rounds”[Mesh] OR round*[tiab])

AND

(“Family”[Mesh] OR “Caregivers”[Mesh] OR “Family Nursing”[Mesh] OR famil*[tiab] OR caregiver*[tiab] OR care giver*[tiab])

AND

("Meta-Analysis" [Publication Type] OR "Systematic Review" [Publication Type] OR systematic review[tiab] OR metaanal*[tiab] OR meta-anal*[tiab] OR systematic[sb])

CINAHL

((MH “Patient Rounds”) OR (TI round*) OR (AB round*))

AND

 ((MH “Family+”) OR (MH “Family Nursing”) OR (MH “Caregivers”) OR (TI famil* OR “care giver*” OR caregiver*) OR (AB famil* OR “care giver*” OR caregiver*))

AND

((MH "Meta Analysis") OR (MH "Systematic Review") OR (TI "Systematic Review") OR (AB "Systematic Review") OR (TI metaanal*) OR (AB metaanal*) OR (TI meta-anal*) OR (AB meta-anal*))

PsycInfo

(TI round* OR AB round*)

AND

((DE “Family” OR DE “Biological Family” OR DE “Dual Careers” OR DE “Dysfunctional Family” OR DE “Extended Family” OR DE “Family Background” OR DE “Family History” OR DE “Family Members” OR DE “Family of Origin” OR DE “Family Relations” OR DE “Family Resemblance” OR DE “Family Structure” OR DE “Family Work Relationship” OR DE “Interethnic Family” OR DE “Interracial Family” OR DE “Military Families” OR DE “Nepotism” OR DE “Nuclear Family” OR DE “Schizophrenogenic Family” OR DE “Stepfamily” OR DE “Caregivers”) OR TI (famil* OR “care giver*” OR caregiver*) OR AB (famil* OR “care giver*” OR caregiver*))

AND

(DE "Systematic Review" OR TI "Systematic Review" OR AB "Systematic Review" OR TI metaanal* OR AB metaanal* OR TI meta-anal* OR AB meta-anal*)

Results 31/3/2022:

PUBMED: 118 results

CINAHL: 78 results

PsycInfo: 11 results

-------------------------------

Total : 207 results

Removing duplicates: 148 Results.

Pubmed: 118 results, 10 included.

CINAHL: 78 results, 3 included (9 with 6 duplicates PUBMED)

PsycInfo: 11 results, 0 included (1 duplicate Pubmed en CINAHL)

Total of 13 included reviews on title and abstract

***4 systematic reviews included in the stystematic Review on full text.***

**9 excluded (3: no family involvement in rounds, 6: no added value outcome FCRs):**

1. Kydonaki K, Takashima M, Mitchell M. Family ward rounds in intensive care: An integrative review of the literature. Int J Nurs Stud. 2021 Jan;113:103771. doi: 10.1016/j.ijnurstu.2020.103771. Epub 2020 Sep 8. PMID: 33080477.

<https://pubmed.ncbi.nlm.nih.gov/33080477/>

*Included*

1. Rea KE, Rao P, Hill E, Saylor KM, Cousino MK. Families' Experiences With Pediatric Family-Centered Rounds: A Systematic Review. Pediatrics. 2018 Mar;141(3):e20171883. doi: 10.1542/peds.2017-1883. Epub 2018 Feb 6. PMID: 29437931.

<https://pubmed.ncbi.nlm.nih.gov/29437931/>

*Included*

1. Cypress BS. Family presence on rounds: a systematic review of literature. Dimens Crit Care Nurs. 2012 Jan-Feb;31(1):53-64. doi: 10.1097/DCC.0b013e31824246dd. PMID: 22156815.

<https://pubmed.ncbi.nlm.nih.gov/22156815/>

*Included*

1. Fernandes AK, Wilson S, Nalin AP, Philip A, Gruber L, Kwizera E, Sydelko BS, Forbis SG, Lauden S. Pediatric Family-Centered Rounds and Humanism: A Systematic Review and Qualitative Meta-analysis. Hosp Pediatr. 2021 Jun;11(6):636-649. doi: 10.1542/hpeds.2020-000240. PMID: 34021029.

<https://pubmed.ncbi.nlm.nih.gov/34021029/>

*Included*

1. Kynoch K, Chang A, Coyer F, McArdle A. The effectiveness of interventions to meet family needs of critically ill patients in an adult intensive care unit: a systematic review update. JBI Database System Rev Implement Rep. 2016 Mar;14(3):181-234. doi: 10.11124/JBISRIR-2016-2477. PMID: 27532144.

<https://pubmed.ncbi.nlm.nih.gov/27532144/>

*Exclusion: No family centered rounds*

*Additional search*: Kynoch K, Chang AM, Coyer F. The effectiveness of interventions to meet family needs of critically ill patients in an adult intensive care unit: a systematic review. JBI Libr Syst Rev. 2011;9(63):2829-2874. doi: 10.11124/01938924-201109630-00001. PMID: 27820310.

<https://pubmed.ncbi.nlm.nih.gov/27820310>

1. Kivelitz L, Schäfer J, Kanat M, Mohr J, Glattacker M, Voigt-Radloff S, Dirmaier J. Patient-Centeredness in Older Adults With Multimorbidity: Results of an Online Expert Delphi Study. Gerontologist. 2021 Sep 13;61(7):1008-1018. doi: 10.1093/geront/gnaa223. PMID: 33388770.

<https://pubmed.ncbi.nlm.nih.gov/33388770/>

*Exclusion: no family involvement*

1. Xyrichis A, Fletcher S, Philippou J, Brearley S, Terblanche M, Rafferty AM. Interventions to promote family member involvement in adult critical care settings: a systematic review. BMJ Open. 2021 Apr 7;11(4):e042556. doi: 10.1136/bmjopen-2020-042556. PMID: 33827833; PMCID: PMC8031009.

<https://pubmed.ncbi.nlm.nih.gov/33827833/>

*Exclusion. Not enough family centered rounds (N=3 van N=20)*

*N*= 3 studies about rounds (Allen et al. 2017, Jacobowski et al. 2010, Weber et al, 2018), also included in systematic review of Kydonaki.

1. Tripodi M, Siano MA, Mandato C, De Anseris AGE, Quitadamo P, Guercio Nuzio S, Siani P, Vajro P. Humanization interventions in general pediatric wards: a systematic review. Eur J Pediatr. 2019 May;178(5):607-622. doi: 10.1007/s00431-019-03370-3. Epub 2019 Apr 4. PMID: 30949888.

<https://pubmed.ncbi.nlm.nih.gov/30949888/>

*Exclusion: No outcome added values of FCR (N=2 FCRs studies included)*

1. Mercedes A, Fairman P, Hogan L, Thomas R, Slyer JT. Effectiveness of structured multidisciplinary rounding in acute care units on length of stay and satisfaction of patients and staff: a quantitative systematic review. JBI Database System Rev Implement Rep. 2016 Jul;14(7):131-68. doi: 10.11124/JBISRIR-2016-003014. PMID: 27532795.

<https://pubmed.ncbi.nlm.nih.gov/27532795/>

*Exclusion: No family involvement*

1. Kiwanuka F, Sak-Dankosky N, Alemayehu YH, Nanyonga RC, Kvist T. The evidence base of nurse-led family interventions for improving family outcomes in adult critical care settings: A mixed method systematic review. Int J Nurs Stud. 2022 Jan;125:104100. doi: 10.1016/j.ijnurstu.2021.104100. Epub 2021 Oct 2. PMID: 34736074; PMCID: PMC8560087.

<https://pubmed.ncbi.nlm.nih.gov/34736074/>

*Exclusion: No focus added value family-centered rounds.*

1. Zamanzadeh V, Orujlu S, Beykmirza R, Ghofrani M. Barriers for nurse participation in multidisciplinary ward rounds: An integrative review. *Nursing Practice Today*. 2021;8(2):96-102. doi:10.18502/npt.v8i2.5120

<https://nlhhg.idm.oclc.org/login?url=https://search.ebscohost.com/login.aspx?direct=true&db=rzh&AN=148176522&login.asp&site=ehost-live>

*Exclusion: No family involvement (only perspective of nurses)*

1. Vetcho S, Cooke M, Ullman AJ. Family-Centred Care in Dedicated Neonatal Units: An Integrative Review of International Perspectives. *Journal of Neonatal Nursing*. 2020;26(2):73-92. doi:10.1016/j.jnn.2019.09.004

<https://nlhhg.idm.oclc.org/login?url=https://search.ebscohost.com/login.aspx?direct=true&db=rzh&AN=142108738&login.asp&site=ehost-live>

*Exclusion: No focus (on added value) rounds*

1. Comp D. Improving Parent Satisfaction by Sharing The Inpatient Daily Plan of Care: An Evidence Review with Implications For Practice and Research. *Pediatric Nursing*. 2011;37(5):237-242. Accessed March 31, 2022.

<https://search-ebscohost-com.nlhhg.idm.oclc.org/login.aspx?direct=true&db=rzh&AN=104637986&login.asp&site=ehost-live>

*Exclusion: No focus on added values FCRs (but the best practice for communicating to parents who are not present during inpatient rounds)*

***4 articles included in the stystematic Review.***

**Additional search**: Registered protocols in Prospero; *terms: Rounds and family*

61 protocols registered, 4 interesting.

1 Study Kydonaki et al. Included

2.Effect of Patient and Family Centred Care interventions for adult intensive care unit patients and their families: A systematic review and meta-analysis No focus on Rounds

Author links open overlay panel[SøsBohart](https://www.sciencedirect.com/science/article/pii/S0964339721001452#!)^[a](https://www.sciencedirect.com/science/article/pii/S0964339721001452#!)^[Ann MereteMøller](https://www.sciencedirect.com/science/article/pii/S0964339721001452#!)^[ab](https://www.sciencedirect.com/science/article/pii/S0964339721001452#!)^[Anne SofieAndreasen](https://www.sciencedirect.com/science/article/pii/S0964339721001452#!)^[a](https://www.sciencedirect.com/science/article/pii/S0964339721001452#!)^[TinaWaldau](https://www.sciencedirect.com/science/article/pii/S0964339721001452#!)^[a](https://www.sciencedirect.com/science/article/pii/S0964339721001452#!)^[CorneliaLamprecht](https://www.sciencedirect.com/science/article/pii/S0964339721001452#!)^[a](https://www.sciencedirect.com/science/article/pii/S0964339721001452#!)^[ThordisThomsen](https://www.sciencedirect.com/science/article/pii/S0964339721001452#!)^[ab](https://www.sciencedirect.com/science/article/pii/S0964339721001452#!)^

3 Lane, D. , Ferri, M. , Lemaire, J. , McLaughlin, K. & Stelfox, H. T. (2013). A Systematic Review of Evidence-Informed Practices for Patient Care Rounds in the ICU*. *Critical Care Medicine, 41*(8), 2015-2029. doi: 10.1097/CCM.0b013e31828a435f. No family

4. Healthcare professionals' experiences of family participation in clinician handovers in intensive care: a qualitative systematic review protocol

*Pauline Wong, Nicole Pope, Andree Gamble, Rose Jaspers, Ruth Endacott Not published*
