# Supplementary material for: The added value of family-centered rounds in the hospital setting: A systematic review of systematic reviews
Source: PLoS One. 2023 Jan 20;18(1):e0280142. doi: 10.1371/journal.pone.0280142 (PMC9858825; doi:10.1371/journal.pone.0280142)
Supplement: Attachment — Submitted filename: REVIEW.pdf [file pone.0280142.s004.pdf]

Review: The added value of family-centered rounds in the hospital setting: A systematic review of systematic reviews

by Woldring et al.

I would like to thank the editor and authors for this interesting paper on the added value of family-centered rounds in the hospital setting. This is an interesting topic with potential impact in clinical practice and for future research.

The design of the study seems sound. A review protocol was registered a priori and PRISMA guidelines were followed. The search and study selection strategy are explained in detail. To increase reliability, the authors selected studies independently and if there was disagreement a third author was involved. Quality appraisal was performed rigorously using the AMSTAR checklist which is used for appraisal of systematic reviews of randomised and non-randomised studies of healthcare interventions. Data extraction was done in a systematic manner. However, I still have some comments on the presentation of the results in this systematic review. My remarks can be found below.

1 - I was wondering, since the aim of this systematic review is to investigate the added value of family-centered rounds without clearly defining 'added value' a priori, this could be a scoping review rather than a classic systematic review. Scoping systematic reviews are done to identify key characteristics or factors related to a concept and it seems that this corresponds with the aim of this study. However, I am not entirely sure that it is a scoping review because this review summarised the effects of FCRs on patient, family member, and healthcare professional outcomes. However, I could not find the actual effect sizes and corresponding levels of uncertainty in this study which is common in a classic systematic review. Could the authors please explain which design they followed and substantiate their decision? Another remark is that PROSPERO registration is not allowed for scoping reviews which could be an additional issue if the authors change the design.

2 - I would remove the sentence: "Four different categories emerged: communication and interaction, situational understanding, inclusion of family in the discussion making process, and relationship within the care situation." from the method section. These are results and therefore do not belong there.

3 - Please cite the excluded studies (n=9) in the second last step of the PRISMA flow chart. This allows the reader to review these excluded studies.

4 - Table 3: Is it possible to provide the total number of participants per included review? A separate column specifying the different outcomes studied would also be helpful. The 'target population' is in my opinion the 'setting' since the population could be healthcare workers, patients and/or family.

5 - In a systematic review of systematic reviews summary results should be presented per included systematic review in a summary of findings table. The authors stated that included

studies were too heterogeneous in their methodology and outcome measures, making it impossible to perform meta-analyses. And I do agree that this is the case here. However, the authors could decide to report effect sizes and corresponding levels of uncertainty per type of outcome for each category (e.g., family member satisfaction) in a table. If necessary, the different definitions of outcomes could be provided next to the results. Moreover, the authors could decide to select studies with a certain level of evidence (e.g. RCTs and/or quasi-experimental research) and provide a table with the respective effect sizes per outcome measure. I think it would be interesting to present some numbers in this systematic review.

6 - p20. The authors state: “However, in the current context of healthcare, with the growing number of care needs and increased complexity of care, the involvement of family caregivers is desirable and necessary. Current healthcare systems are increasingly relying on family members to take on care responsibilities. This is also true for a wide variety of adult patients and not strictly for the most seriously ill, unresponsive ones or those without decision-making capacity [69].”

Yes, this is true. However, isn’t an increased reliance on family members because of shortages in professional caregivers a negative evolution? The authors should nuance their message here or at least provide some more explanation. I do not think that the argument is entirely correct, and this statement is repeated in the conclusion (In the light of the current challenges in healthcare, FCRs seem to be highly relevant for adult non-critical care patients as well.)

7 - An important limitation of this review is the limited focus on the added value of FCRs. The authors acknowledge this in the discussion and mention that implementation should be studied. However, could this be more substantiated with relevant literature?
