# Supplementary material for: The added value of family-centered rounds in the hospital setting: A systematic review of systematic reviews
Source: PLoS One. 2023 Jan 20;18(1):e0280142. doi: 10.1371/journal.pone.0280142 (PMC9858825; doi:10.1371/journal.pone.0280142)
Supplement: Attachment — Submitted filename: Respons to the reviewers.docx [file pone.0280142.s003.docx]

Dear Academic editor,

Thank you for your careful review of our manuscript on the added values of family-centered rounds.

We welcome your constructive criticism and have improved the manuscript accordingly.

In the table below you find a response to each comment raised by the editor and/or reviewer.

| *Point of the editor and reviewer* | *Response of the authors* |
| --- | --- |
| **Method** |  |
| Lines 107-108 you state that outcomes, benefits and effects were used in the single systematic reviews. Can you, in response to reviewers, state which reviews these were? In the manuscript, you should cite these reviews or change the text to be more generic, ie away from specific reviews. You may want to consider moving the definition of added value to the end of the introduction and incorporate into the objective where the concept is first introduced in the text. | We agree with this comment, and your suggestion. We have moved the introduction of the concept of ‘added value’ to the end of the introduction. |
| Line 110 is missing punctuation after the [15.16] citations. | Amended |
| In the study selection please state the number of agreements/disagreements that required adjudication by a third author. | There were four records with no agreement between the two researchers. We have clarified this in the article.  *There were four records with no agreement. Disagreement was solved through discussion with a third author (WP).* |
| In the data extraction and synthesis section it’s unclear what the process for extraction and synthesis was, particularly “included systematic reviews were systematically and independently reviewed by two reviewers” (lines 149-150). What does ‘reviewed’ mean in the context of data extraction? Did you use any software to support data extraction (eg Microsoft Excel)? Was a particular data extraction tool used? If so, how was the tool created / extraction headings selected? How were disagreements between the two reviewers addressed at point of extraction? Which two authors performed the extraction and synthesis? | Thank you for this comment. We have expanded the description of the extraction and synthesis process.  *The research team developed a table with variables to be extracted from the selected reviews in order to guide the data extraction process. Two reviewers (JMW and MLL) read each review carefully and extracted the following data into the table for comparison: authors, country, research question, target population, number of studies included, design of the included studies and the results regarding the added values of FCRs from the perspectives of patients, families, and healthcare professionals. Once the data were extracted and grouped, the research team discussed the data and synthesized categories based on the findings with regard to ‘added values’. Four different categories emerged: communication and interaction, situational understanding, inclusion of family in the discussion making process, and relationship within the care situation*. |
| **Results** |  |
| Figure 1 uses an outdated version of the PRISMA diagram, please update it to the current version. | The PRISMA diagram is updated to the current version.  See figure 1 Flowchart |
| You state that out of 91 studies included in the four reviews, there were 24 duplications. Do you know why there were so few (relatively) duplicates? Eg did the reviews have different search criteria or research questions? (this may be more relevant to reflect upon in the discussion than results) | The limited number of duplicates is mainly due to the fact that the reviews had different target populations (Rea and Fernandes focused on pediatrics and Kydonaki on adult critical care). The reviews of Rea et al. and Fernandes et al. have pediatrics as the target population and have most duplications (14). Kydonaki had the focus on adult critical care and therefore showed no duplications with Rea et al. and Fernandes et al. This is described in the study Characteristics. |
| The sentence starting on line 197, “These reviews included 28…” is hard to follow, please rephrase. | The sentence is rephrased as:  *The review of Rea et al. included 28 and the review of Fernandes et al. 29 single studies in pediatric (non)-critical care with 14 duplicates between these two reviews [23,24].* |
| Line 201, “performed in the past 4[sic] years” would perhaps be better phrased as “performed in the latter four years of the study. Note that 2014-2018 is a five year period (14, 15, 16, 17, 18) – either amend to a latter five years or change to 2015-2018, whichever is accurate. | The sentence is rephrased as:  *Their review included 15 single studies; the majority (12) performed in the latter five years of the study.* |
| There are typos in the results, please proof read the full paper though I’ve tried to spot some.   - Line 254, Kydonaki needs to be capitalised. - Line 264, “these” should be “this” and the sentence should continue immediately after the previous sentence. | The entire research team has read the paper again.  Typos are amended |
| **Discussion** |  |
| The third paragraph starting on line 316, “The findings of our study…” would be better placed at the end of the first paragraph. | We agree and have replaced this line, as suggested, to the first paragraph. |
| You say that meta analysis wasn’t possible, but how do you know when you didn’t assess the individual studies yourself? Do you mean a meta-analysis of the systematic reviews’ data? This should be made clearer, as well as the potential and/or uncertainty around whether a meta-analysis of individual studies would be possible. Also consider making it clear that the judgement of studies being highly heterogeneous wasn’t yours (as you didn’t assess this for individual studies) but rather from the reviews themselves. | Amended to:  *The authors of the four systematic reviews concluded that the studies included in their review were too heterogeneous in their methodology and outcome measures, making it impossible to perform meta-analyses.* |
| You state that almost all studies were in paediatric settings or critical care settings; please state how many (as you’ve done for adult non-critical care settings). | Amended to:  *FCRs have almost exclusively been studied from the perspective of families and/or healthcare professionals, but not patients. This is due to the fact that almost all studies have been conducted either in the pediatric (non)-critical care setting (51) or in the adult critical care setting (15). In the former group parents or guardians are the decision makers because of the young age of the patient population whereas in the critical care setting families are surrogate decision makers as patients are unresponsive most of the time.* |
| Line 323, “1” should be “one” (please check manuscript for all numbers of ten or less). | Amended |
| Line 325, children being “under-age” appears colloquial. Perhaps just remove this and leave it as “cannot take decisions on their own”. I also think that you should engage with debate about whether children can or cannot make informed decisions about their care as you appear to uncritically accept the statement that they cannot take decisions on their own. There’s an increasing body of work examining paediatric shared decision making in a broader context, though I’m unsure whether this has yet reached person centred ward rounds (may be an opportunity to better suggest it). Your final paragraph before the limitations begins to touch on this but not specifically in relation to paediatric involvement. | Thank you for this important comment. We are aware of the debate whether children can or cannot make informed decisions. There exists no universal agreement as to at what age minors should be deemed decision-making competent, as this clearly depends on their maturity. As described in the final paragraph we see patient, family and healthcare professionals as equal partners in discussion and decision making. We described it broadly because, as described in the introduction, our focus is on the adult (non-critical care) patient.  In this paragraph, we wanted to make clear that children below 18 years of age are in most western countries by law not entitled to make decisions on their own, but depending on their maturity may be increasingly involved from age 12 and beyond.  Amended  *This can be understood from the idea that children below 18 years of age are in most western countries by law not entitled to make decisions on their own, so parents or guardians are automatically involved, whereas in the critical care setting families are surrogate decision makers most of the time.* |
| Line 334-335, please rephrase “highlighted positively” as it’s unclear. | Amended:  *The results derived from studies in the pediatric and critical care setting indicate added values for patients, families and healthcare professionals.* |
| Your final limitation, with only two of 67 studies being in Europe, arguably isn’t a limitation but rather one of the key findings from this review, that person centred ward rounds are yet to be properly studied in countries outside of the US. I think you should make more of this in your broader discussion rather than leaving it as a limitation. I understand it’s a grey area between being a finding and a limitation because only including papers written in English may have introduced this bias, but the general lack of English speaking countries (eg UK, Australia, Ireland) suggests that this is problem with either a) spread / adoption, or b) terminology (are there different terms used in other English speaking countries?). It would be good for you to consider and reflect upon these in the discussion, either earlier or if you decide to leave this in the limitations. | We agree that only two studies in Europe also is a finding from this review so we added the following in the discussion:  *It is remarkable that only two of the 67 included single studies were conducted in Europe.* *Most research was executed in the USA and Canada, which might suggest that FCRs are not yet widely adopted and does not seem a substantial subject for scientific studies outside the Angelo-Saxon countries.*  We do not think there is a problem with terminology. We have done a broad search on rounds with Mesh terms etc. and found a lot of different rounds. It seems unlikely that we missed studies due to search terms.  Limitations:  *The finding that most research was executed in the USA and Canada, limiting the generalizability of the findings to the context of these countries. Research is hence required in the European context to establish the feasibility and added value in this specific healthcare context.* |
|  |  |
