# Supplementary material for: The added value of family-centered rounds in the hospital setting: A systematic review of systematic reviews
Source: PLoS One. 2023 Jan 20;18(1):e0280142. doi: 10.1371/journal.pone.0280142 (PMC9858825; doi:10.1371/journal.pone.0280142)
Supplement: Attachment — Submitted filename: Response to Reviewers.docx [file pone.0280142.s005.docx]

Dear Academic editor and reviewer,

Thank you for your careful review of our manuscript on the added values of family-centered rounds.

We welcome your constructive criticism and have improved the manuscript accordingly.

In the table below you find a response to each comment raised by the reviewer.

| *Point of the editor and reviewer* | *Response of the authors* |
| --- | --- |
| 1 - I was wondering, since the aim of this systematic review is to investigate the added value of family-centered rounds without clearly defining ‘added value’ a priori, this could be a scoping review rather than a classic systematic review. Scoping systematic reviews are done to identify key characteristics or factors related to a concept and it seems that this corresponds with the aim of this study. However, I am not entirely sure that it is a scoping review because this review summarised the effects of FCRs on patient, family member, and healthcare professional outcomes. However, I could not find the actual effect sizes and corresponding levels of uncertainty in this study which is common in a classic systematic review. Could the authors please explain which design they followed and substantiate their decision? Another remark is that PROSPERO registration is not allowed for scoping reviews which could be an additional issue if the authors change the design. | *The objective of this systematic review of systematic reviews is to summarize the evidence regarding the added value of FCRs from the perspectives of patients, family, and healthcare professionals.*  We agree with the reviewer that the concept of 'added value' is not unambiguously interpreted in the various studies included. We have considered presenting a certain one-dimensional definition in advance. Ultimately, we did not do this in order to do justice to the interpretations in the various studies. Nevertheless, we believe that the current methodical, systematic approach and the questioning justifies the interpretation of a systematic review. In our review it was not the main goal to discuss characteristics of the concept ‘added value’ (Indeed, in that case a scoping review could be more opportune). In our review a clinically meaningful question was chosen as a focus, in which a systematic review seems to be the best fitting approach. i.e. *The added value of family-centered rounds in the hospital setting;* not specifically the analysis of the conceptual specifications, antecedents or theoretical representations of ‘added value’.  Indeed, the most traditional form of a systematic review has not been chosen here, which only includes effect studies that refer to double-blind randomized studies and associated effect measures. However, we believe that the chosen diversity in included designs does not have to be a major objection to abandoning the systematic method.  It was our choice to look for a synthesis at the level of ‘systematic reviews’ and not primarily at the independent studies included in it. In our opinion it is therefore more logical to stick to the 'systematic review' design.  We have therefore chosen to state the estimated level of evidence in the studies; something that also belongs to a systematic review.  We hope to have provided sufficient argumentation to continue with a systematic review. |
| 2 - I would remove the sentence: “Four different categories emerged: communication and interaction, situational understanding, inclusion of family in the discussion making process, and relationship within the care situation.” from the method section. These are results and therefore do not belong there. | *Thank you for this comment. We have removed it from the method section.* |
| 3.Please cite the excluded studies (n=9) in the second last step of the PRISMA flow chart. This allows the reader to review these excluded studies. | *We have added the nine excluded studies as supplementary material and referenced in the result section:*  *Result section:*  These nine publications were excluded, ultimately resulting in four systematic reviews eligible for inclusion. *Appendix search strategy* |
| 4 - Table 3: Is it possible to provide the total number of participants per included review? A separate column specifying the different outcomes studied would also be helpful. The ‘target population’ is in my opinion the ‘setting’ since the population could be healthcare workers, patients and/or family. | Thank you for these points.  We agree that ‘Setting’ is a better name and amended *Target population to Setting.*  We have also added a separate column with the different outcomes studied.  We agree with the reviewer that in certain cases it may indeed be opportune and desirable to enumerate samples in the synthesis of systematic reviews. We have also considered this before, but concluded that the included designs differed to such an extent that an addition could give an unsuitable result. In other words, there could even be participants in (semi)qualitative designs that would be added to quantitative designs. To counteract this bias, we have chosen to present only the size of the sample per included study (systematic review) in a table (see table 3). |
| 5 - In a systematic review of systematic reviews summary results should be presented per included systematic review in a summary of findings table. The authors stated that included studies were too heterogeneous in their methodology and outcome measures, making it impossible to perform meta-analyses. And I do agree that this is the case here. However, the authors could decide to report effect sizes and corresponding levels of uncertainty per type of outcome for each category (e.g., family member satisfaction) in a table. If necessary, the different definitions of outcomes could be provided next to the results. Moreover, the authors could decide to select studies with a certain level of evidence (e.g. RCTs and/or quasi-experimental research) and provide a table with the respective effect sizes per outcome measure. I think it would be interesting to present some numbers in this systematic review. | We thank the reviewer for this important point of view. We have set the suggestion given here as our goal before. We thought we might be able to present clarifying forest plots as well. However, in line with the previous remark, this turned out not to be possible because of the too great chance of bias in the comparison of the samples and the methods used. |
| 6 - p20. The authors state: “However, in the current context of healthcare, with the growing number of care needs and increased complexity of care, the involvement of family caregivers is desirable and necessary. Current healthcare systems are increasingly relying on family members to take on care responsibilities. This is also true for a wide variety of adult patients and not strictly for the most seriously ill, unresponsive ones or those without decision-making capacity [69].” Yes, this is true. However, isn’t an increased reliance on family members because of shortages in professional caregivers a negative evolution? The authors should nuance their message here or at least provide some more explanation. I do not think that the argument is entirely correct, and this statement is repeated in the conclusion (In the light of the current challenges in healthcare, FCRs seem to be highly relevant for adult non-critical care patients as well.) | Discussion  *However, in the current context of healthcare, with the growing number of care needs and increased complexity of care, the involvement of family caregivers is desirable and necessary. Current healthcare systems are increasingly relying on family members to take on care responsibilities. This is also true for a wide variety of adult patients and not strictly for the most seriously ill, unresponsive ones or those without decision-making capacity [69].”*  The sentence is rephrased as:  Family members are the major source of support for patients during hospitalization and at home. Therefore, family involvement is essential to ensure quality and continuity of care. Furthermore, in the current context of healthcare, with the growing number of care needs and increased complexity of care, the involvement of family caregivers becomes even more desirable and necessary. This is true for a wide variety of adult patients and not strictly for the most seriously ill, unresponsive ones or those without decision-making capacity.  Conclusion  *In the light of the current challenges in healthcare, FCRs seem to be highly relevant for adult non-critical care patients as well.*  Rephrased as:  Since family is the major source of support for patients and current healthcare more and more relies on the support of family, FCRs seem to be highly relevant for adult non-critical care patients as well. |
| 7 - An important limitation of this review is the limited focus on the added value of FCRs. The authors acknowledge this in the discussion and mention that implementation should be studied. However, could this be more substantiated with relevant literature? | *Furthermore, the organization and education of FCRs are important aspects of implementation and should be studied.*  Amended to:  Furthermore, the organization and education of FCRs are important aspects of implementation*.* A structure or protocol with clear roles of FCRs participants should be studied and developed [71,72]. |
